# Transplantation of a quaternary structure neutralizing antibody epitope from dengue virus serotype 3 into serotype 4

**DOI:** 10.1038/s41598-017-17355-5

**Published:** 2017-12-07

**Authors:** Douglas G. Widman, Ellen Young, Usha Nivarthi, Jesica A. Swanstrom, Scott R. Royal, Boyd L. Yount, Kari Debbink, Matthew Begley, Stephanie Marcet, Anna Durbin, Aravinda M. de Silva, William B. Messer, Ralph S. Baric

**Affiliations:** 1 Department of Epidemiology, School of Public Health, University of North Carolina, Chapel Hill, NC USA; 2 Department of Microbiology and Immunology, University of North Carolina School of Medicine, Chapel Hill, NC USA; 3 Center for Immunization Research, Department for International Health, Johns Hopkins Bloomberg School of Public Health, Baltimore, Maryland USA; 4 Present Address: Department of Natural Sciences, Bowie State University, Bowie, MD USA; 5 Present Address: Department of Molecular Microbiology and Immunology, Oregon Health and Science University, Portland, OR USA

## Abstract

Dengue vaccine trials have revealed deficits in our understanding of the mechanisms of protective immunity, demonstrating a need to measure epitope-specific antibody responses against each DENV serotype. HmAb 5J7 binds to a complex, 3-monomer spanning quaternary epitope in the DENV3 envelope (E) protein, but it is unclear whether all interactions are needed for neutralization. Structure guided design and reverse genetics were used to sequentially transplant larger portions of the DENV3-specific 5J7 mAb epitope into dengue virus serotype 4 (DENV4). We observed complete binding and neutralization only when the entire 3 monomer spanning epitope was transplanted into DENV4, providing empirical proof that cooperative monomer-hmAb 5J7 interactions maximize activity. The rDENV4/3 virus containing the most expanded 5J7 epitope was also significantly more sensitive than WT DENV4 to neutralization by DENV3 primary immune sera. We conclude that the hinge-spanning region of the 5J7 quaternary epitope is a target for serotype-specific neutralizing antibodies after DENV3 infection.

## Introduction

The four serotypes of dengue viruses (DENV1-4) are estimated to cause around 100 million cases of dengue fever or dengue hemorrhagic fever each year^1^. As exemplified by the highly successful, yellow fever virus (YFV) 17D vaccine developed in the early 1930’s and more recently Japanese encephalitis virus (JEV), vaccination is a feasible strategy for preventing and controlling mosquito-borne flavivirus infections^2–4^. In other flavivirus infections where neutralizing antibody titers >10 protect^5,6^, similar titers in DENV infection are complicated by the existence of four heterotypic serotypes and heterotypic cross neutralization. While the presence of neutralizing antibodies has been long considered a correlate of protection for flaviviruses, recent data from dengue vaccine trials prove that the presence of antibodies that neutralize DENVs in cell culture do not necessarily confer protection^7^. New assays and reagents are needed to characterize human antibody responses to dengue virus infections and vaccination and to identify requirements for protection beyond mere neutralizing antibodies.

A major challenge to DENV vaccine development is the existence of 4 serotypes and the need for vaccines to confer protection against all 4 serotypes. With an approximate 60% amino acid divergence between the E proteins of the 4 serotypes, immunity to one serotype usually does not confer long-lasting cross-protective immunity to the other serotypes^8^. Additionally, people experiencing a secondary DENV infection with a new serotype face a greater risk of progression to severe DHF (Dengue hemorrhagic fever) and DSS (Dengue shock syndrome). Severe disease is a result of immunopathology, likely mediated by aberrant T cells^9^ and non-neutralizing antibodies induced by the first infection. Moreover, pre-existing antibodies may increase viral load in secondary infections through the process of antibody-dependent enhancement (ADE) of infection of Fc receptor bearing cells^10^. As such, a successful DENV vaccine should ideally elicit robust anti-DENV protective immunity against all 4 serotypes to prevent subsequent dengue illness, especially severe illness that can result from ADE infection. To date this has been a difficult challenge to overcome, especially in those seronegative at the time of vaccination.

It has been known since the early 1980s through passive transfer experiments that antibodies targeting the E glycoprotein can protect from lethal flavivirus challenge^11^. Structural studies with human monoclonal antibodies (hmAbs) isolated from dengue patients have provided high resolution maps of epitopes on the viral surface. These studies have also led to the development of new tools and reagents to identify correlates and mechanisms of protective immunity following natural infection or vaccination^12–16^.

**Figure 1:**
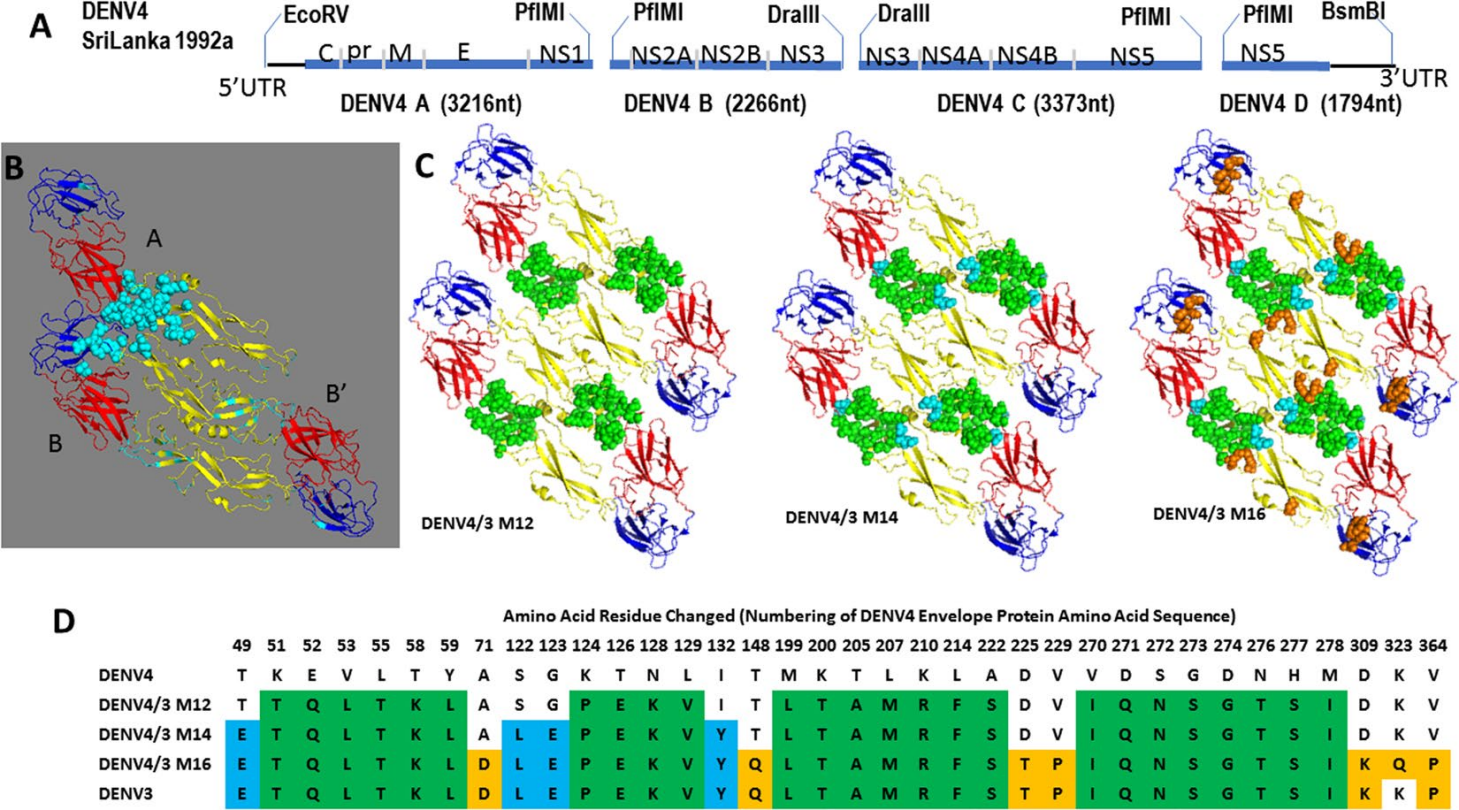
Design of infectious cDNA clones of DENV3 and generation of recombinant DENV4/3 viruses. (**A**) Genome schematic of DENV4 infectious clone design including restriction endonucleases used to generate subgenomic fragments from individual high-copy plasmids. Size of subgenomic fragments indicates positions in DENV genome where breaks were made to circumvent bacterial instability and toxicity. **(B)** Amino acid residues of a single 5J7 epitope are shown teal balls. In each ribbon structure of E protein, Domain 1 is red, Domain 2 is yellow and Domain 3 is blue. Residues of the 5J7 epitope spread across all three monomers (B, B’ form a dimer, A is in the adjoining monomer) are teal. PDB accession code 4CBF(DENV4). **(C)** Four E monomers in a partial raft are shown for each 5J7 transplant with increasingly larger transplants into DENV4. Transplanted amino acids in each of the DENV4 chimeras can be seen as highlighted AA; DENV4 M12 (green residues), DENV4 M14 (green + cyan residues), and DENV4 M16 (green + cyan + orange residues). **(D)** The chart shows the amino acids changed in DENV4 E protein by transplantation of DENV3 amino acid sequences to generate DENV4 M12, M14, and M16 respectively. Amino acid number represents residue from start of DENV4 E protein. The K323Q mutation in DENV4 M16 is a tissue culture adaptation and is included in the orange balls in B. Colors correspond to highlighted AA residues in **(C)**.

In DENV, the E proteins are arranged in 3 sets of parallel homo-dimers, which form a raft. Thirty rafts cover the surface of the particle and represent primary targets for neutralizing antibody^14^. Our group and others have characterized DENV-specific antibodies in people exposed to natural and experimental infections or live attenuated vaccines^13,17,18^. Both serotype-specific and cross-reactive strongly neutralizing mAbs have been isolated from the memory B cells of donors with a history of primary and secondary DENV infections^17,19,20^. The location of mAb epitopes on the envelope glycoprotein often, but not always, differs between serotypes and commonly the paratope recognizes a complex quaternary epitope that is present only on fully assembled and intact virions. While the structural footprints of several human neutralizing mAbs on the viral envelope have been determined through the use of cryo-electron microscopy, the resolution of these structural studies can only predict the relative contribution of different fractions of the quaternary epitope to monoclonal and/or polyclonal antibody neutralization phenotypes. Recently, DENV3-specific potently neutralizing antibody, 5J7^17^, was identified as using a complex quaternary epitope to neutralize DENV3 in a manner previously hypothesized for West Nile virus (WNV)^21,22^. Importantly, the 5J7 monoclonal antibody footprint interacts with distinct residues encoded in 3 adjacent monomers, A, B’ and B (Fig. 1B), localized within two dimers of the raft. However, the relative contribution, if any, of each monomer to 5J7 binding and neutralization activity is unknown.

We have previously described reverse-genetics systems for all 4 DENV serotypes^13,23,24^, and have used these clones to transplant entire domains^13^ or defined antibody epitopes^24^ between serotypes. The chimeric recombinant viruses have been used to study properties of human immune sera and mAbs isolated from dengue patients. By transplantation of a portion of the 5J7 epitope contained within the hinge region of a single monomer of DENV1, we achieved a partial binding and neutralization of the recombinant DENV1 by hmAb 5J7^25^. Here, we describe the properties of a panel of recombinant DENV4 strains capturing increasingly larger footprints of the complex 5J7 epitope, eventually encompassing all of the key footprint residues on three different monomers. We observed an epitope size-dependent effect on 5J7 binding and neutralization. Importantly the recombinant virus with a fully reconstructed quaternary epitope that spanned across three E monomers in the raft not only demonstrated the greatest sensitivity to 5J7 mAb binding and neutralization, but also captured a large fraction of the polyclonal DENV3 type specific neutralizing antibody responses in many people infected with this serotype. To our knowledge, this is the first successful example of exchanging a complex, 3-monomer spanning quaternary epitope between virus strains, paving the way for similar structure-guided immunogen design in other viruses or on protein scaffolds. Our results firmly establish the utility of epitope transplant recombinant DENVs for functional mapping of mAb epitopes and for measuring epitope specific responses in human immune sera and for the development of bivalent DENV vaccine candidates.

## Results

### Transplantation of a type-specific neutralizing DENV3 mAb epitope into DENV4

We previously described the generation and characterization of a reverse genetics system for production of recombinant DENV3 (rDENV3)^23^. Utilizing similar techniques, we have also developed a cDNA infectious clone (IC) system for a clinical isolate of DENV4 isolated in Sri Lanka in 1992 and belonging to genogroup I^26^. To circumvent instability and toxicity during bacterial plasmid amplification in *E coli*, the DENV4 genome was subcloned into 4 distinct fragments (Fig. 1A). Naturally occurring class IIs restriction endonuclease sites in the DENV4 genome at nucleotide position 3216 (PflMI; recognition sequence: CCAAACAGTGG), 5482 (DraIII; CACCAGGTG), and 8855 (PflMI; CCAGATTTTGG) were utilized to divide the genomic cDNA. Additionally, an EcoRV site in the bacterial vector and upstream of a T7 promoter sequence was used to generate the 5′ end of the genome, while a BsmBI site in the vector was used for the 3′ genomic end (Fig. 1A). DENV4 recovered from using the infectious clone system was indistinguishable from the wild type parental virus with respect to cell culture replication and surface antibody epitope display^13^.

Human monoclonal antibody (hmAb) 5J7 is a strongly neutralizing serotype specific antibody isolated from a patient who recovered from a DENV3 infection^17^. Using cryo-electron microscopy, the foot print of hmAb 5J7 was recently mapped to a quaternary epitope that spans 3 adjacent E protein molecules on the viral envelope^15^ (Fig. 1B). We recently reported that transplantation of the central core of the epitope on a single E monomer (similar to the transplant seen in DENV4/3 M12 in Fig. 1C) into DENV1 resulted in a partial gain in binding and neutralization of the recombinant virus by 5J7^25^, highlighting the potential importance of contact resides on adjacent E molecules B and B’ (Fig. 1B) for hmAb 5J7 binding and neutralization. Attempts to place larger portions of the 5J7 epitope into DENV1 proved unsuccessful. Consequently, we switched to the DENV4 reverse genetic platform to transplant progressively increasing sections of the hmAb 5J7 epitope, starting from the core epitope on a single E molecule, to the complete foot print spanning all three E molecules (Fig. 1). Using escape mutations as a guide and the cryo EM structure^15,27^, the envelope domain I/II (EDI/II) hinge region was superimposed upon the DENV3 E protein crystal structure to approximate the footprint of an antibody epitope binding region. Following amino acid (AA) and nucleotide alignments between DENV3 and DENV4, interserotypic variant AA were identified between DENV3 and DENV4 (Fig. 1C,D). Three distinct recombinant DENV were generated, each containing sequentially additional DENV3 residues and thus transplanting larger epitope footprints that eventually encompassed residues encoded in the A, B’ and B monomers (Fig. 1C,D). Nucleotide sequences in the DENV4 IC were modified to facilitate AA changes to match DENV3, and subgenomic cDNAs capturing these changes were synthesized commercially (BioBasic; Amherst, NY). Recombinant DENV4 were generated as described above, and recovered chimeric viruses used for phenotypic characterization. These recombinant DENV4 strains have been designated DENV4/3 M12, DENV4/3 M14, and DENV4/3 M16, representing progressively increased portions of DENV3 5J7 epitope displayed on DENV4 (Fig. 1C,D). DENV4/3 M16 encompasses the entire complex quaternary epitope of 5J7 spread over 3 monomers of E protein^15^; interestingly, during isolation and passage in Vero cells a mutation emerged at position K323Q (Fig. 1).

### rDENV4/3 viruses are viable and demonstrate distinct fitness characteristics in cell culture

**Figure 2:**
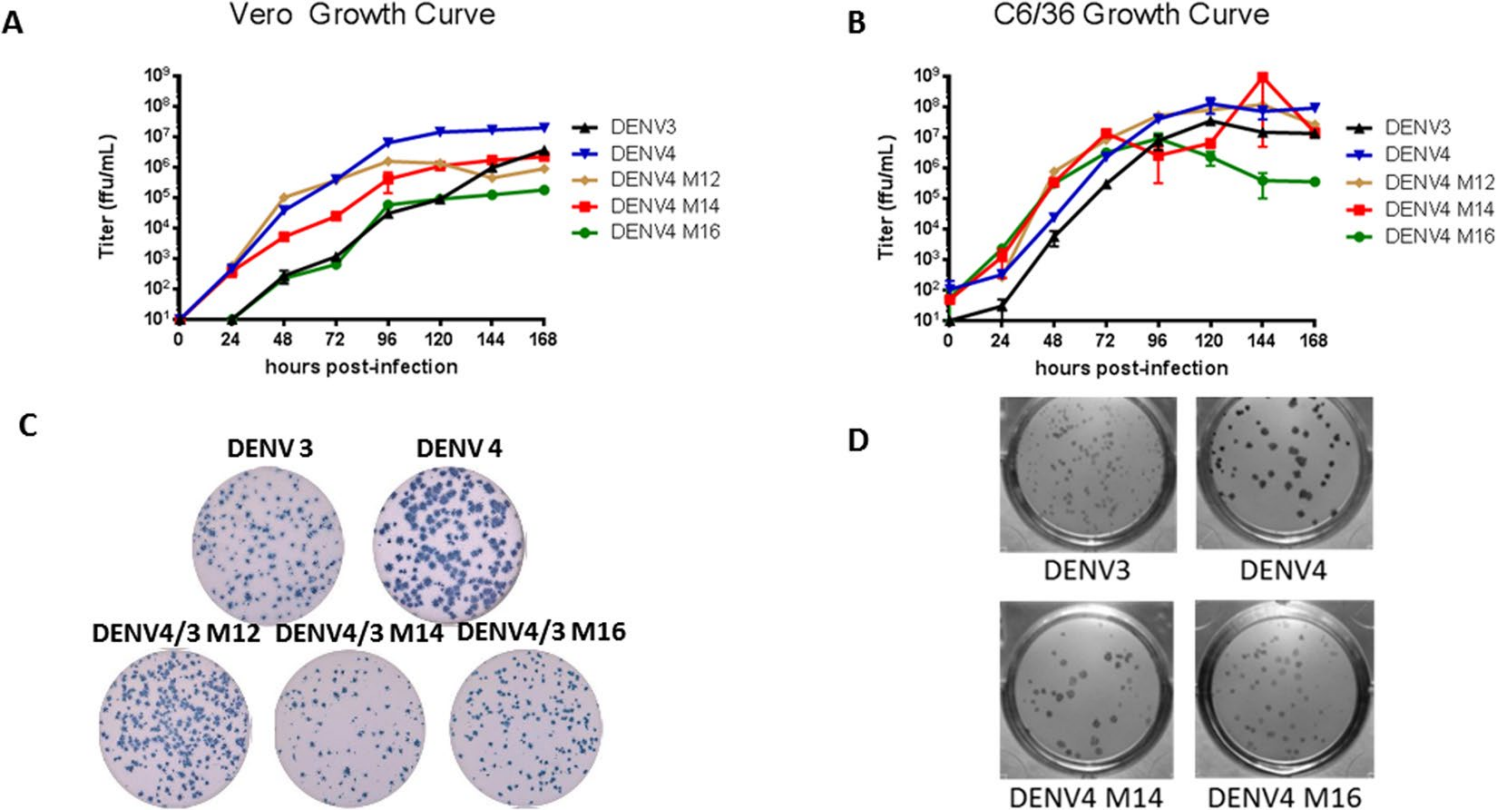
Growth kinetics of rDENV4/3. (**A**) DENV3, DENV4, DENV4 M12, DENV4 M14, and DENV4 M16 were used to perform multi-step growth curves on Vero-81 **(A)** and C6/36 **(B)** cells. Infectious titers are presented as ffu/ml in the cell culture supernatant at the indicated time point post-infection. Examples of foci for the same virus panel on Vero **(C)** or C6/36 **(D)** cells.

All three of the rDENV4/3 viruses (M12, M14, and M16) were successfully recovered following electroporation and subsequent passage on C6/36 cells. Using sequence analyses and RT-PCR RFLP analyses, all viral stocks were pure and contained only the rDENV of interest (Supplemental Fig. 1). Growth kinetics in C6/36 cells and Vero-81 cells were slightly attenuated compared to that of the parental WT DENV4 IC, but not parental WT DENV3 although M16 had the lowest final titer in both cell types (Fig. 2A and B). Correspondingly the rDENVs also formed smaller foci compared to their DENV4 WT parent and more similar to their DENV3 WT parent (Fig. 2C and D). These results indicate that rDENV4 viruses containing transplanted regions of DENV3 are viable and demonstrate suitable growth characteristics for further characterization.

### Gain of binding to 5J7 mAb

**Figure 3:**
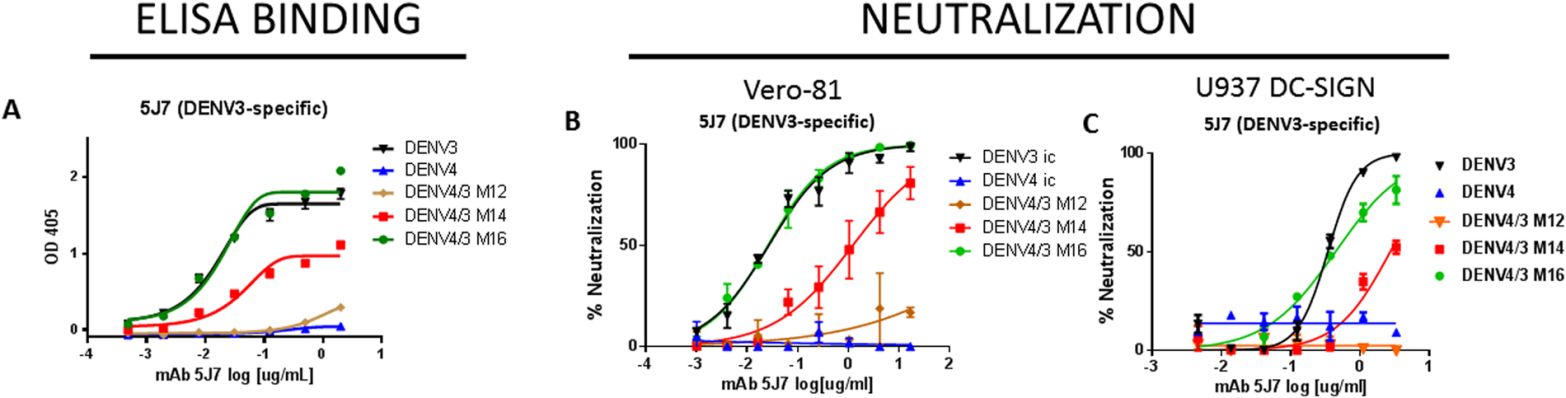
Gain of binding and neutralization of hmAb 5J7 on rDENV4/3. (**A**) ELISA binding curves for DENV3-specific hmAb 5J7 on DENV3, DENV4, DENV4 M12, DENV4 M14, and DENV4 M16. Neutralization assays on Vero-81 **(B)** and U937-DC-SIGN **(C)** for DENV3, DENV4, DENV4 M12, M14, and M16. Error bars represent the range of values. Graphs show one representative experiment of at least 2 independent experiments.

To determine the impact of increasing the size of the transplanted region on hmAb 5J7 binding and neutralization, we performed binding and neutralization assays using the recombinant viruses and hmAb 5J7. The hmAb demonstrated progressively better binding to DENV4/3 M12, M14 and M16 viruses, respectively (Fig. 3A). Importantly, binding to the DENV4/3 M16 virus was comparable to WT DENV3, providing empirical support for structural studies that predicted that all three monomers were critical for 5J7 binding. These data also indicate that no more than 4 AA (the difference in transplanted AA between DENV4/3 M12 and M14) were responsible for conferring improved 5J7 binding to DENV4/3 M14. Subsequently, adding 7 more AA on monomers A, B’ and B achieved complete gain of binding of 5J7. We also performed neutralization assays with the recombinant viruses and hmAb 5J7. When the assays were performed on Vero cells, we observed efficient neutralization of M16 that was indistinguishable from DENV3, moderate neutralization of M14 and no neutralization of the M12 virus (Fig. 3B). We obtained similar results when human U937 cells expressing DC-SIGN (an attachment receptor for DENV) were used for the assay (Fig. 3C). Importantly, DENV4/3 M16 was the most sensitive to 5J7 neutralization activity, supporting binding data and structural analyses, validating the role of the entire quaternary structure in binding and neutralization.

### DENV4 epitopes and envelope structure

**Figure 4:**
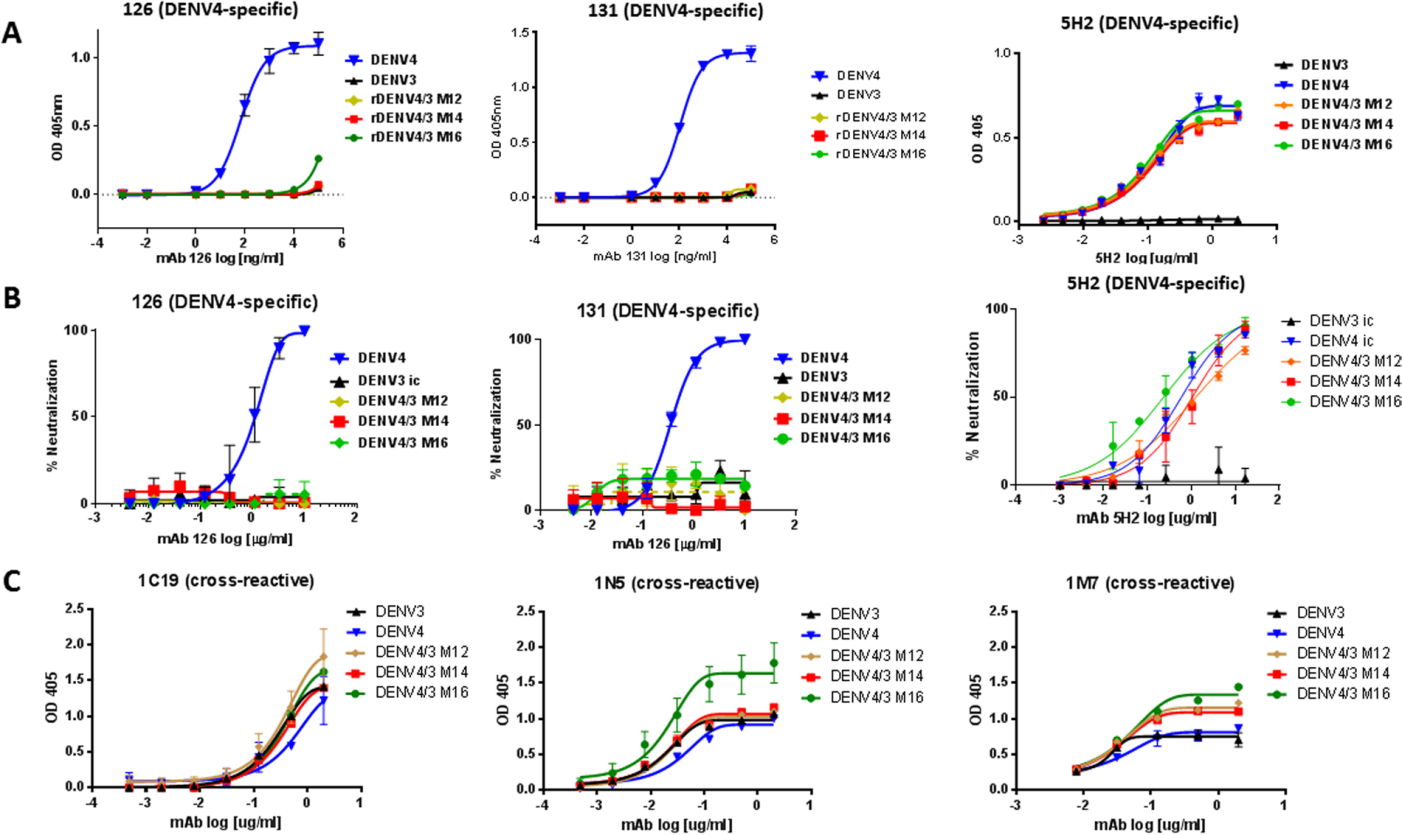
Neutralization and binding of DENV4-specific and cross-reactive monoclonal antibodies on rDENV4/3 panel (A) ELISA binding curves of DENV3, DENV4, DENV4/3 M12, DENV4/3 M14 and DENV4/3 M16 by DENV4-specific mAbs show loss of 126, 131 DENV4 epitopes in the chimeric viruses but not the 5H2 DENV4 epitope. **(B)** U937-DC-SIGN Neutralization assays with these same DENV4-specific mAbs also show loss of 126, 131 DENV4 epitopes while 5H2 was retained. **(C)** ELISA binding curves with cross-reactive mAbs that are distal to the 5J7 epitope show similar binding to all viruses. Error bars represent the range of values. Graphs show one representative experiment of at least 2 independent experiments.

**Figure 5:**
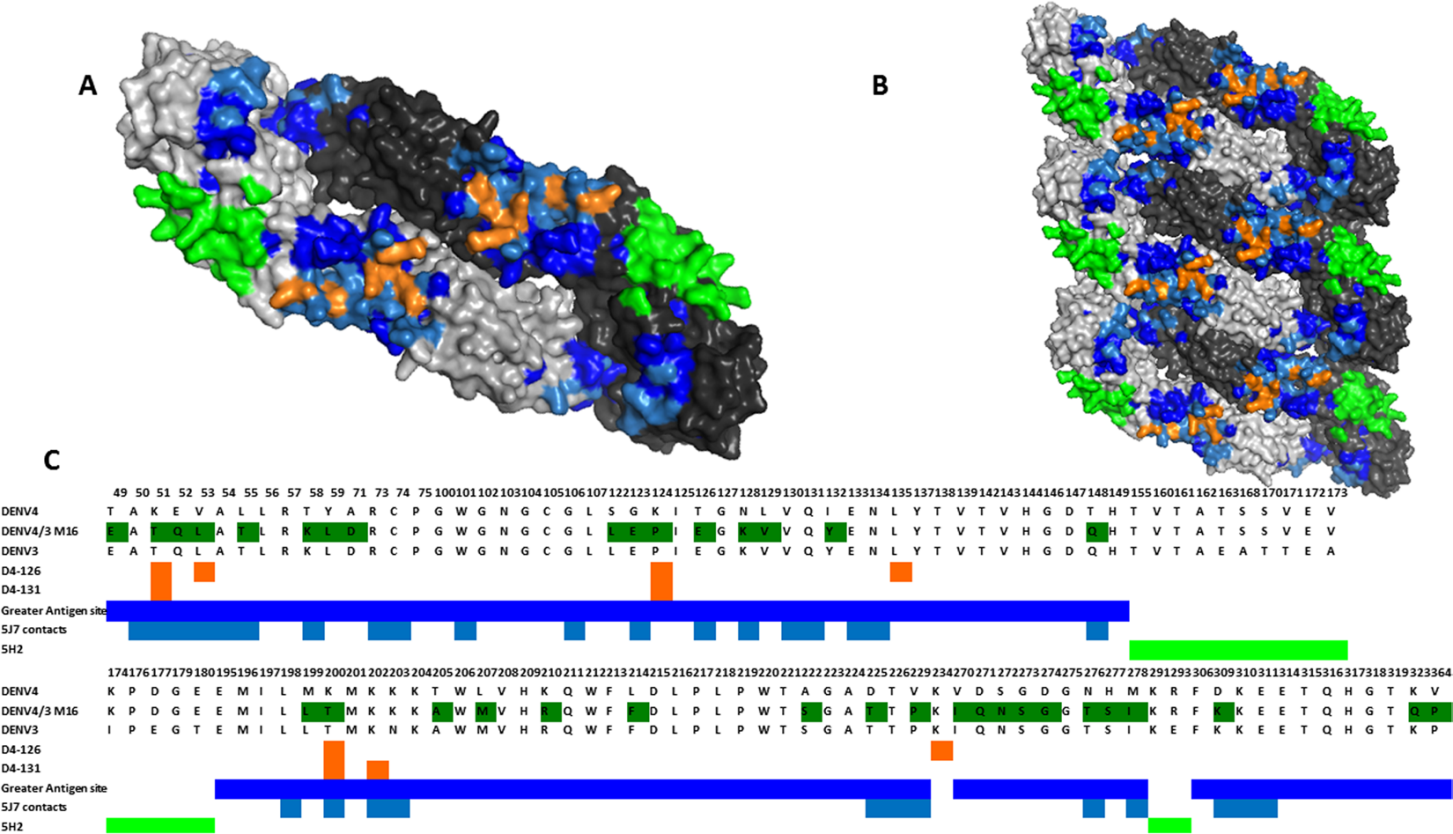
Dengue 4/3 E-glycoprotein dimer (**A**) and raft of 3 dimers (**B**) are shown. Contact residues for 5J7 mAb are shown in light blue, while greater antigenic area residues of M16 construct (transplanted and homologous) are shown in dark blue. The greater antigenic area includes residues that are homologous in both DENV 3 and DENV4 and can be seen to spread across multiple dimers. Orange residues have previously been shown to be important for binding of DENV4-specific hmAbs 126 and 131^28^ and overlap with known 5J7 contact residues. Binding residues for DENV4-specific mAb 5H2, shown in green^58^, do not overlap the 5J7 epitope. (**C**) Amino acid key to DENV E-glycoprotein. AA changes to DENV4 E protein to generate DENV4 M16 are shown in dark green as well as contact residue of DENV3-specific mAbs 5J7 and DENV4-specific mAbs 126, 131 and 5H2. Amino acid number represents residue from start of DENV4 E protein. Colors correspond to highlighted AA residues in **(A** and **B)**.

We tested 3 DENV4-specific mAbs, hmAbs 126 and 131, which have overlapping epitopes near the hinge region of EDII, and chimpanzee mAb 5H2, which binds to EDI (Fig. 4A and B). Nivarthi et. al. previously demonstrated that 126 and 131 did not bind or neutralize rDENV4/3 M12 because the transplanted 5J7 epitope sits in and around the same region of E protein that would be bound by 126 and 131 (Fig. 5)^19^. We confirmed that the DENV4/3 M14 and M16 behave in a similar manner and do not bind hmAbs 126 and 131^28^. Conversely, 5H2, which recognizes a DENV4 epitope on EDI directly adjacent to the 5J7 footprint, bound and efficiently neutralized the panel of three DENV 4/3 viruses (Fig. 4A and B). Thus, regions of E protein adjacent to the transplant appear undisturbed during 5J7 epitope exchange.

The envelope structures of the WT parental and rDENV4/3 virus panel were evaluated using mAbs known to bind to regions of the E protein distal to the portion that we transplanted from DENV3. As shown in Fig. 4B and C, all mAbs recognizing epitopes that have been mapped to distal sites from the 5J7 epitope (5H2, 1C19, 1M7 and 1N5), bound similarly to the rDENV4/3 virus panel, WT DENV3 and WT DENV 4, although rDENV4/3 M16 appears to display similar binding affinities but slightly increased peak binding levels of 1N5 and to a lesser extent 1M7. These results suggest that the overall global virion architecture of the rDENV4/3 panel appears to be minimally altered by transplantation of DENV3 AA residues into DENV4.

### rDENV4/3 neutralization by DENV3 immune sera

**Figure 6:**
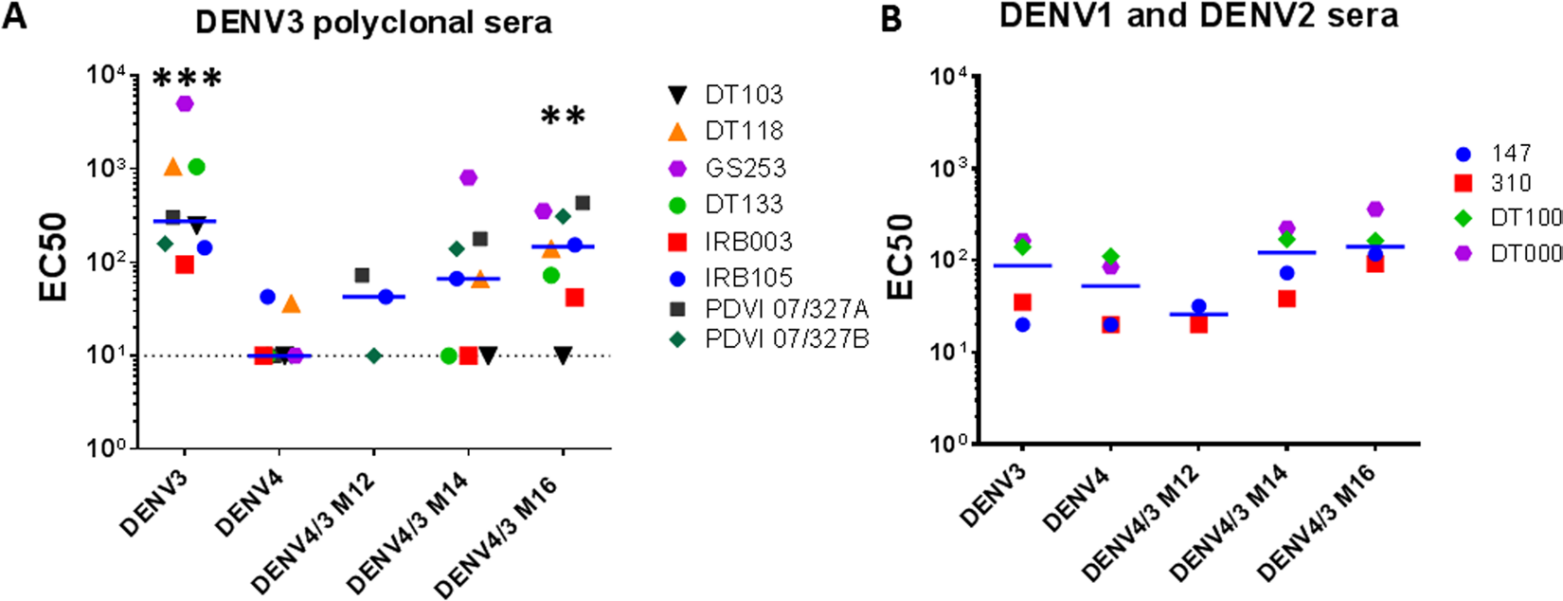
Polyclonal DENV immune sera neutralization. The rDENV4/3 and parental WT viruses were subjected to virus neutralization tests using the flow cytometry-based U-937-DC-SIGN neutralization assay. EC50s are shown for each serum. **(A)** Sera used for neutralization was collected from convalescent travelers with a history of a single DENV3. DENV4/3 M12, M14 and M16 are increasingly sensitive to DENV3 sera neutralization at levels comparable to WT DENV3 IC, indicating that these travelers mounted DENV3-specific immune responses directed at the transplanted DENV3 epitope region. EC50s for DENV4 were significantly different from DENV3 (p = 0.0078) and DENV4/3 M16 (p = 0.0156) but not significantly different from M14 (p = 0.0625) or M12 (p = 0.5000) by a 2-way Wilcoxon matched-pairs rank test. EC50s for DENV4/3 M16 were not significantly different from DENV3 (p = 0.1953) by the same test. **(B)** Heterotypic serum neutralization. Sera collected from human donors recovered from primary DENV1 and DENV2 infections were assayed against DENV3, DENV4, DENV4/3 M12, DENV4/3 M14, and DENV4/3 M16 on U937 + DC-SIGN cells to assess sensitivity to non-specific humoral immune responses. EC50s were not significantly different by a 2-way Wilcoxon matched-pairs rank test. Compared to DENV4, p = 0.125 for M14 and p = 0.125 for M16. Compared to DENV3, p = 0.125 for M14 and p = 0.125 for M16. Neutralization curves for all sera are shown in Supplementary Figures 2 and 3.

Using the panel of rDENV4/3 M12, M14 and M16 viruses, we next assessed if the DENV3 5J7 epitopes displayed on DENV4 were targets of serum neutralizing antibodies after recovery from primary DENV3 infections. rDENV4/3 M14 and rDENV4/3 M16 were more sensitive to neutralization by primary DENV3 immune sera compared to WT DENV4 demonstrating the presence of serum antibodies that track with the 5J7 epitope in DENV3 immune sera (p = 0.02 by one-way Anova with Dunn’s multiple comparisons test, adjusted p = 0.0073 for DENV4/3 M16) (Fig. 6A). In fact, five and 7 of the 8 subjects tested had DENV3 neutralizing antibodies that tracked with DENV4/3 M14 and DENV4/3 M16 viruses, respectively. These results underscore the importance of the entire 5J7 epitope as a target of human antibodies that neutralize DENV3. Across individuals, variable levels of the DENV3 response was captured by the 5J7 epitope, suggesting that additional epitopes are targets of DENV3 neutralizing antibodies. It is also possible natural variation across DENV3 strains within the 5J7 epitope accounts for some neutralizing antibodies that failed to track with the recombinant viruses^23^. As DENV4/3 M16 captured the largest fraction of neutralizing antibodies compared to the other recombinant viruses, we conclude that residues in all 3-monomers are required for the most efficient capture of 5J7-like responses.

### rDENV4/3 polyclonal neutralization sensitivity is specific

To eliminate the possibility that the panel of rDENV4/3 viruses had been modified in such a way as to increase their sensitivity to neutralization by DENV immune sera in a non-specific manner, neutralization assays were performed with immune sera collected at late convalescence (>6 months) from subjects exposed to a primary DENV2 (DT100) or DENV1 (310 and 147) infection and secondary DENV infections (DT 000). These immune sera cross-neutralized DENV3, DENV4 and DENV4/3 M14 and M16 in U937 + DC-DIGN cells to similar degrees and were not significantly different by a 2-way Wilcoxon matched-pairs rank test. (Fig. 6B) indicating that the gain of DENV3 neutralization sensitivity in the DENV4 background is specific for DENV3 and not the result of global structural changes that make the rDENV panel more sensitive to neutralization by heterotypic sera.

## Discussion

Human mAbs that bind quaternary epitopes on flaviviruses were first described for WNV followed by the 4 DENV serotypes^21,22,14,15,20^. Some of these quaternary structure-dependent epitopes are present on the E dimer but some are only present on the intact virion and may span across dimers to bind to neighboring monomers/dimers, as is seen with the 5J7 epitope. It is becoming apparent that knowing the epitope specificity of an antibody response to a vaccine may be essential for understanding the correlates of DENV protective immunity. Large-scale clinical trials of new vaccine candidates have demonstrated that *in vitro* gold standard neutralization results do not always predict vaccine efficacy, current data indicate that the standard Vero neutralization assay without regard to epitope specificity does not always correlate with protective immunity^7,29,30^. This is not the first time that failure of *in vitro* characteristics, previously small-plaque and temperature-sensitive phenotypes failed to be predictive of vaccine efficacy^31–33^. Measuring epitope specificity of polyclonal sera is a daunting task, although recent advances in elucidating the contributions of individual hmAb to the overall serological repertoire have demonstrated that only a few neutralizing antibodies may dominate a protective response against influenza virus^34,35^. While this approach provides rich molecular information on an individual basis, new reagents are needed for population based studies. Single and multiple mutations in the E protein have been effectively used to measure type specific antibodies in human polyclonal sera by loss of neutralization^36^. Using epitope-transplanted chimeric DENV viruses, such as the set described herein, gain of neutralization can be measured to easily and accurately measure epitope specific antibody binding and neutralization responses in complex polyclonal sera. Such chimeric viruses may be combined with the 4 serotypes of DENV or they may be used in combination with antigen depletion of sera to measure epitope specificity of polyclonal sera.

Here, we show incremental gain of binding of the 5J7 monoclonal with the increasing size of the transplanted epitope in both binding and neutralization assays. It is also abundantly clear that additional residues in adjoining monomers significantly enhanced 5J7 binding to DENV4/3 M16 virus, clearly demonstrating that residues on A, B’ and B monomers are critically important for 5J7 recognition, maximal neutralization and quaternary epitope function. More importantly, we show that the larger epitope transplants that include residues of 3 different E proteins can be used to measure the amount of antibody that targets this antigenic site in DENV 3 primary immune polyclonal sera. Because individual monoclonal antibodies like 5J7 can only infer the location of the primary neutralizing antigenic site that is recognized by the polyclonal response, epitope transplant provides a means of demonstrating the importance of the A, B’ and B monomers in the global polyclonal neutralization response against DENV3. We do not always capture the entire DENV3 neutralization titer present in the polyclonal sera, suggesting that additional neutralizing sites and/or genetic variability within the epitope must exist in DENV3, supporting the critical need for identifying and charactering more type specific hmAb for each DENV serotype. Importantly, our data argue that our rDENV 4/3 panel is a powerful tool for measuring DENV3 epitope-specific antibodies in polyclonal sera. Indeed, the DENV4/3 M14 and M16 have very recently been successfully used to track development of antibodies to the 5J7 epitope in a large scale DENV3 cohort study with similar results^37^.

A variety of approaches are being investigated to present complex epitopes in virus-like particles and on scaffolds, both for diagnostic and vaccine applications^38–41^. To date, none have presented complex quaternary epitopes that span multiple monomers. We have previously demonstrated that residues in the A monomer of the 5J7 epitope can be transplanted into DENV1^25^. The transplant of the DENV3 5J7 epitope into DENV 4 is unique from previous transplants in that this is the first transplanted epitope in any virus in which residues of adjoining monomers and dimers were also modified to capture an entire complex quaternary epitope. We detected a clear gain of epitope specific responses to 5J7 in the DENV4/3 recombinant viruses as the size of the transplanted region was increased to capture residues adjacent in B and B’ E molecules. With the DENV4/3 panel we have enlarged the scope and complexity of epitopes that can be successfully transplanted, effectively reconstructing a functional epitope that spans multiple monomers in the E glycoprotein raft^15^. In the DENV4/3 M16 recombinant virus, it is interesting that a single nucleotide change emerged in cell culture and that some minor differences were noted in the peak binding levels of broadly cross reactive fusion loop antibodies. DENV4/2 M16 displayed higher peak binding to 1M7 and 1N5 fusion loop antibodies, perhaps reflecting different maturation states and prM levels on virions. Future studies will focus on unraveling the role of this mutation and its phenotypes in epitope exchange viruses.

The advent of modern molecular techniques and genomics has allowed for a new, highly-rational approach to be taken in the development of tools for epitope analysis and of new vaccine candidates^42^. New developments in reverse genetics can be utilized to generate infectious clones for a number of viruses, including flaviviruses^12,43–50^. Importantly, quaternary epitopes and complex conformational neutralizing epitopes exist in many highly evolving and variable viruses, thus the methods described herein should be applicable to broad, based immunogen design for multiple pathogens. Live virus vaccines for yellow fever virus and more recently Dengue viruses provide new avenues for improving vaccine immunogenicity and safety, using reverse genetic approaches. In particular, the DENV tetravalent vaccine usually elicits immune responses against all four serotypes, regardless of manufacturer^29,51–54^. Nonetheless, the scope of the response varies based on vaccine serotype and the pre-immune status of the host. A detailed understanding of the location and functional activity of conformational and quaternary epitopes in vaccine performance offers novel opportunities for developing epitope specific correlates of protective immunity, engineered serotypes for improved vaccine immunogenicity and performance, and simplified bivalent vaccine formulations. (reviewed in^51,55^).

## Methods

### Cell lines and viruses

Vero cells (ATCC# CCL-81), C6/36 cells (ATCC CRL-1660) and DC-SIGN-expressing U937 cells (ATCC CRL-1593.2 transfected with a retrovirus coding human DC-SIGN) and their growth conditions have been previously described^13^ The rDENV3 clone, based on a Sri Lankan 1989 strain, was initially described in detail^23^ and the DENV4 clone was based on the Sri Lankan 1992a sequence^26^ and has only briefly been described previously^13^.

### Ethics statement

Convalescent DENV immune blood samples were collected from travelers visiting countries where dengue is endemic. Our protocol for collecting dengue immune blood samples was approved by the Institutional Review Board of the University of North Carolina at Chapel Hill (protocol 08-0895). Written informed consent was obtained from all subjects before their participation in the study. Studies at UNC and NIH were performed in accordance with the relevant guidelines and regulations^56,57^.

### DENV immune sera

DENV serum samples used in this study were obtained from the existing dengue traveler collection as mentioned above. The samples were not from acute clinical patients and hence were not PCR confirmed. All samples were coded and analyzed anonymously.

### Generation of the rDENV4/3Panel

To generate the 3 different size rDENV4/3 viruses (M12, M14, and M16), increasing numbers of amino acid residue sequences were transplanted into DENV4 from DENV3. As a result of our quadripartite infectious clone design^13,23,24^, all changes were isolated to the A fragment of the DENV4 clone genome. The 3 increasing sizes of the 5J7 epitope transplant were synthesized (BioBasic, Buffalo, NY) and incorporated into 3 different DENV4 fully assembled DNA genomes, transcribed, and then the genome length RNAs electroporated into Vero-81 cells to generate the rDENV4/3 panel of viruses, all 3 of which were viable. The first attempt at DENV4/3 M16 acquired a tissue culture adaptation, Env K323Q. The DENV4/3 M16 described in this paper was rederived with this additional Env K323Q residue and is otherwise isogenic with DENV4. Recombinant viruses were subjected to full length sequencing to demonstrate the presence of appropriate subsets of mutations as previously described^13^.

### Vero Cell Titration/Focus Assays

For viral titrations, viral stocks were diluted 10-fold serially in dilution medium (OptiMEM, Grand Island, NY) supplemented with 2% heat-inactivated fetal bovine serum (HI-FBS; Hyclone Defined) and 1x antibiotic/antimycotic. Following a 1hr infection, cells and inoculum were overlaid with overlay medium: OptiMem (Gibco, Grand Island, NY) containing 5% carboxymethylcellulose, 2% HI-FBS (Hyclone defined), and 1x antibiotic/antibiotic (Gibco, Grand Island, NY). Following a 4–6 day incubation (to achieve countable foci of infection), cell monolayers were extensively washed with 1xPBS followed by fixation in a solution of 80% methanol:20% phosphate-buffered saline (PBS) (v:v). Fixed cell monolayers were blocked in a solution of PBS containing 5% non-fat milk, and incubated with a primary antibody cocktail of 4G2 and 2H2 murine mAbs. Following thorough PBS washes, infectious foci were visualized using HRP-goat-anti-mouse secondary antibody (KPL, Gaithersburg, MD), followed by TrueBlue substrate and foci were enumerated and used to calculate infectious titer.

### Growth Curves

DENV3, DENV4, and the rDENV4/3 panel were subjected to multi-step (MOI = 0.1) growth curve analyses on Vero or C6/36 cells. Briefly, Vero or C6/36 cell monolayers in 12-well plates were inoculated with indicated virus at an MOI = 0.1. At 24hr intervals, all medium was removed and an equivalent amount of fresh medium was added. Supernatants were clarified by centrifugation and stored at −80 °C until viral titrations were performed. Virus grown on Vero cells was titrated on Vero cells, and C6/63-derived virus was titrated on C6/36 cells, enumerated, and plotted over time to generate growth curves.

### Vero Cell Neutralization Assays

Neutralization on Vero-81 cells has previously been described^13^. Briefly, monolayers of Vero-81 cells in 24 well plated were infected with a virus:antibody/serum cocktail that had previously incubated for 1hr at 37 °C to allow for Ab:virion binding. Following a 1hr incubation at 37 °C for infection, cells and inoculum were overlaid with overlay medium (see above). Following a 4–6 day incubation at 37 °C, monolayers were fixed, stained, and geometric mean neutralization titers enumerated using wells containing no antibody as a control for infection.

### U-937-DC-SIGN Titration and Neutralization Assay

Human sera or monoclonal antibodies were serially diluted 3-fold and mixed with sufficient virus to cause 15% infection in U937 + DC-SIGN cells. Dilution media contained reduced (2%) fetal bovine serum. The virus:antibody mixtures were incubated for 45 minutes in a 96-well plate at 37 °C. Following this incubation, 5 × 10^4^ cells were added and the infection was allowed to proceed for 2 hours at 37 °C. Unbound virus was washed with infection media and the volume of media in each well was increased to 200 μl, and the cells were returned to 37 °C for a total of 24 hours. After 24 hours, the cells were washed with PBS, fixed in paraformaldehyde, permeabilized with saponin, blocked with normal mouse serum in permeabilization buffer, and stained with AlexaFluor 488-conjugated (Molecular Probes, Eugene, OR) 2H2 antibody. Unbound antibody was washed off, and cells were resuspended in Hank’s Buffered Salt Solution (Gibco, Grand Island, NY) supplemented with 2% fetal bovine serum. Assays were performed twice and in duplicate. Samples were read on a Guava easyCyte 5HT flow cytometer (Millipore) as previously described by our group^27^. All serum neutralization curves are shown in Supplementary Figures 2 and 3.

### Reverse Transcriptase-PCR (RT-PCR) and Restriction Fragment Length Polymorphism (RFLP)

In order to confirm our recombinant virus stocks were pure and did not contain any WT DENV, highly sensitive RT-PCR and RFLP were performed as previously described^13^. Briefly, RNA isolated from viral stocks, DNase treated (Turbo DNase, Ambion, Carlsbad, CA), was reverse transcribed and the resulting cDNA amplified (Qiagen One Step RT-PCR Kit, Germantown, MD) using primers specific for each serotype. Positive controls from WT DENV RNA were run for each reaction as was a no template control. Presence of amplicons for rDENV4 and no other rDENV indicates absence of any species of DENV1-3.

For RFLP, RNA isolated from viral stocks was converted to cDNA and the portion of the E protein gene containing the transplanted epitope was amplified by PCR. The resulting amplicon was gel purified on a 1% agarose gel, and subjected to specific restriction endonuclease (RE) digests designed to cut the WT amplicon, but not the rDENV amplicon (due to ablation of the RE site by epitope transplantation). While the approximately 1000 bp wild-type amplicon was cut by BsiWI into an 800 bp and 200 bp fragments, the rDENV were not digested, indicating the absence of WT DENV4 in the recombinant viral stocks.

### Statistical Assays

Geometric mean neutralization titers were determined by 4-way non-parametric curve fitting with upper and lower bounds of 100 and 0. Nonparametric column statistic (2-way Wilcoxon matched-pairs rank test, 2-tailed Mann-Whitney test, and 1-way ANOVA Friedman test with adjusted p value calculated by Dunn’s test) were used to calculate p-values. All statistics were calculated using Graphpad Prism (La Jolla, CA).

## Electronic supplementary material

Supplementary Information
