# Supplementary material for: Transplantation of a quaternary structure neutralizing antibody epitope from dengue virus serotype 3 into serotype 4
Source: Sci Rep. 2017 Dec 7;7:17169. doi: 10.1038/s41598-017-17355-5 (PMC5719398; doi:10.1038/s41598-017-17355-5)
Supplement: Supplementary file 1 — Supplementary Information [file 41598_2017_17355_MOESM1_ESM.pdf]

Supplemental Data for

Transplantation of a quaternary structure neutralizing antibody epitope from dengue virus serotype 3 into serotype 4

Douglas G. Widman[1], Ellen Young[1], Usha Nivarthi[2], Jesica A. Swanstrom[1], Scott R. Royal[1], Boyd L. Yount [1], Kari Debbink[2,,¥], Matthew Begley[1], Stephanie Marcet[1], Anna Durbin[δ], Aravinda M. de Silva[2], William B. Messer[1,§], Ralph S. Baric[1,2,*]

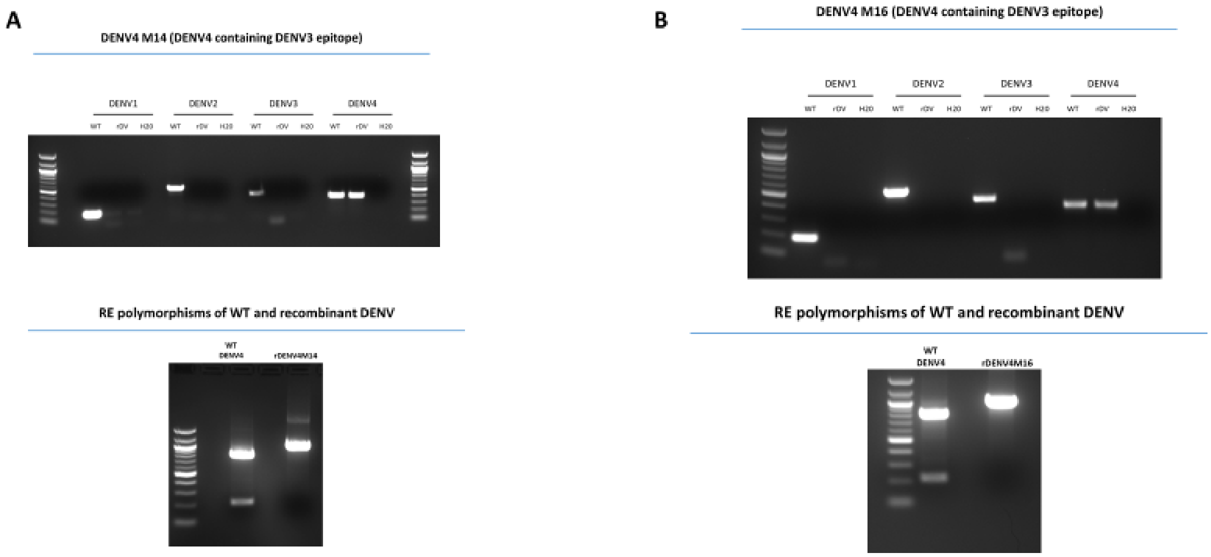

Supplementary Figure 1. Confirmation of rDENV stock purity. (A) From viral culture supernatant, total RNA was isolated and DNAse treated. The RNA was used as templates for individual serotype-specific PCR amplification. From left to right for each serotype: positive control using RNA from a characterized WT DENV stock, serotype-specific amplicon for the rDENV being tested. In this case both rDENV4/3 are on a DENV4 backbone, as indicated by the

DENV4 amplicon. **(B)** RFLP on amplicons generated using PCR primers that span the transplanted epitope of DENV3. This transplantation disrupted the recognition site of BsiWI, and thus only the WT amplicon is digested, indicating there is no WT DENV4 present in the viral stocks.

Supplemental Table 1

			EC50 Neut. titre in U937					M16	
SerumA4:H17D4 A4:H15AA4:H17	Source	Past infection	DENV3	DENV4	DENV4/3 M12	DENV4/3 M14	DENV4/3 M16	% Gain of DV3 neut.	CONCLUSION
DT 103	Nicaragua, 1995	Primary DENV3	248	<20		<20	<20	0	No Gain of DV3 neutralization
DT 118	Nicaragua, 2009	Primary DENV3	1049	36		66	139	13	Partial gain of DV3 neut in M16
GS0263	LIAI	Primary DENV3	5006	<20		806	354	7	Partial gain of DV3 neut in M14 and M16
DT 133	Columbia, S.A. 2002	Primary DENV3	1058	<20		<20	73	7	No Gain of DV3 neutralization
IRB003	Thailand 2001	Primary DENV3	95	<20		<20	42	44	Gain of DV3 neut in M16
IRB105	Thailand, 2002	Primary DENV3	144	43	43	67	154	100	Gain of DV3 neut in M16
PDVI 07/327A	PDVI	Primary DENV3	303	<20	73	178	434	100	Gain of DV3 neut in M14 and 16
PDVI 07/327B	PDVI	Primary DENV3	158	<20	<20	139	311	100	Gain of DV3 neut in M14 and 16
DT001	Sri Lanka 1996; D2 isolated	Primary DENV2	140.1	110.8		169.7	164.2		No change in neutralization
147	Bolivia, 2012	Primary DENV1	20	20	32	73	117		increase in neutralization
310	PDVI	Primary DENV1	35	20	20	38	91		No change in neutralization
DT000	Sri Lanka	secondary	164	85		223	361		No change in neutralization

Gain of neutralization was calculated by dividing the EC50 of the chimeric virus by the EC50 of DENV3 and multiplying by 100.

Supplementary Table 1. Summary of polyclonal human sera used in studies. Table of sera used in the reported study. Data included (from left to right) includes location and date collected, neutralization titers of each serum sample against the panel of rDENV4/3 viruses in U937+DC-SIGN cells, analysis of serum samples and the epitope-specific proportion of neutralization against each rDENV. Gain was calculated by dividing the EC50 of the chimeric virus by the EC50 of DENV3 and multiplying by 100.

Primary DENV 3 Sera

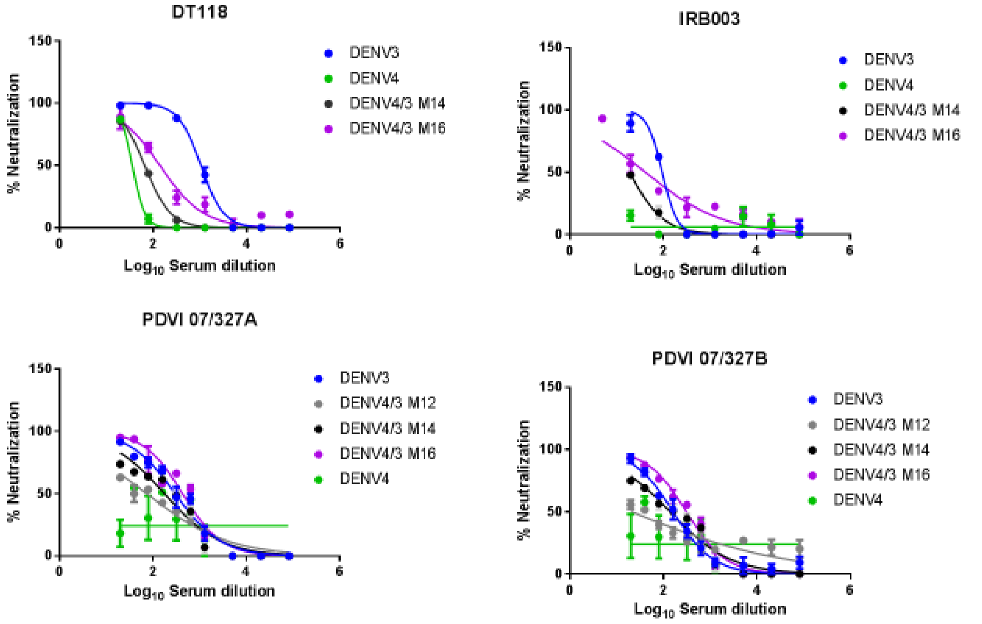

Primary DENV 3 Sera

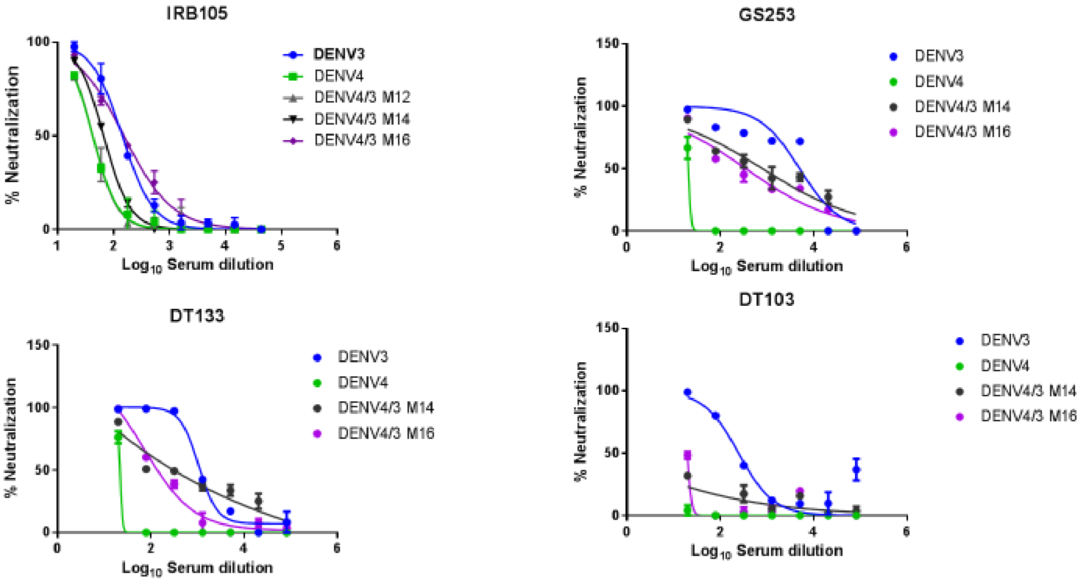

Supplimentary Figure 2. Titration curves for all of DENV3 poly clonal sera neutralization of DENV3, DENV4, DENV4/3 M14, DENV4/3 M16 and sometimes DENV4/3 M12.

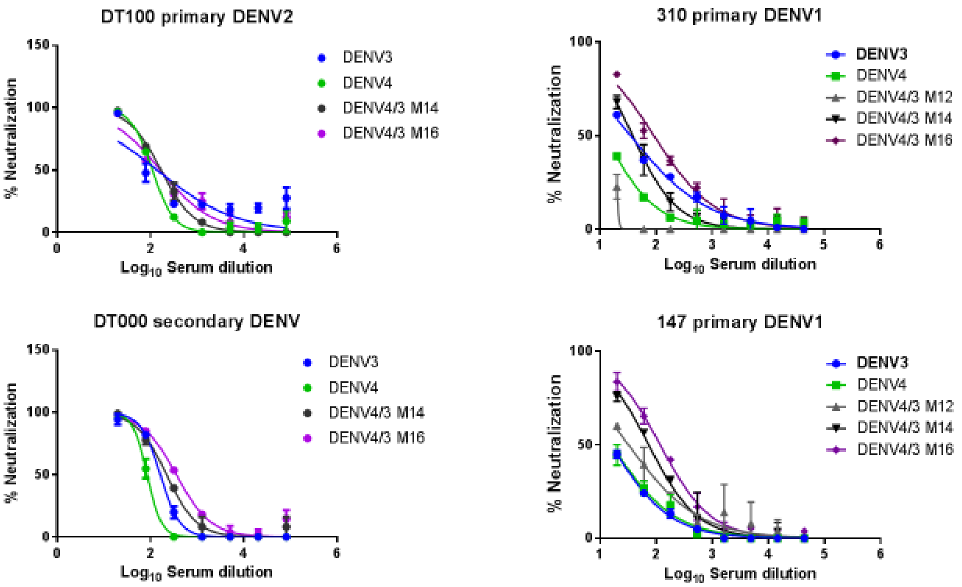

Supplimentary Figure 3. Titration curves for primary polyclonal sera neutralization of DENV3, DENV4, DENV4/3 M14, DENV4/3 M16 and sometimes DENV4/3 M12.
